# Lower High-Density Lipoproteins Levels During Human Immunodeficiency Virus Type 1 Infection Are Associated With Increased Inflammatory Markers and Disease Progression

**DOI:** 10.3389/fimmu.2018.01350

**Published:** 2018-06-14

**Authors:** Damariz Marín-Palma, Gustavo A. Castro, Jaiberth A. Cardona-Arias, Silvio Urcuqui-Inchima, Juan C. Hernandez

**Affiliations:** ^1^Infettare, Facultad de Medicina, Universidad Cooperativa de Colombia, Medellín, Colombia; ^2^Grupo Inmunovirologia, Facultad de Medicina, Universidad de Antioquia, UdeA, Medellín, Colombia; ^3^Escuela de Microbiología, Universidad de Antioquia, UdeA, Medellín, Colombia

**Keywords:** human immunodeficiency virus type 1, high-density lipoproteins, inflammation, inflammasomes, C-reactive protein, NLRP3, CD4+ T-cell count, acquired immunodeficiency syndrome

## Abstract

**Introduction:**

High-density lipoproteins (HDL) are responsible for the efflux and transport of cholesterol from peripheral tissues to the liver. In addition, HDL can modulate various immunological mechanisms, including the inflammatory response. Inflammasomes are multiprotein complexes that have been reported to be activated during human immunodeficiency virus type 1 (HIV-1) infection, thus contributing to immune hyperactivation, which is the main pathogenic mechanism of HIV-1 progression. However, the relationship between HDL and inflammasomes in the context of HIV-1 infection is unclear. Therefore, this research aims to explore the association between HDL and the components of the inflammatory response during HIV-1 infection.

**Methodology:**

A cross-sectional study, including 36 HIV-1-infected individuals without antiretroviral treatment and 36 healthy controls matched by sex and age, was conducted. Viral load, CD4+ T-cell counts, serum HDL, and C-reactive protein (CRP) were quantified. Serum cytokine levels, including IL-1β, IL-6, and IL-18, were assessed by ELISA. The inflammasome-related genes in peripheral blood mononuclear cells were determined by quantitative real-time PCR.

**Results:**

HIV-1-infected individuals showed a significant decrease in HDL levels, particularly those subjects with higher viral load and lower CD4+ T-cell counts. Moreover, upregulation of inflammasome-related genes (NLRP3, AIM2, ASC, IL-1β, and IL-18) was observed, notably in those HIV-1-infected individuals with higher viral loads (above 5,000 copies/mL). Serum levels of IL-6 and CRP were also elevated in HIV-1-infected individuals. Significant negative correlations between HDL and the mRNA of NLRP3, AIM2, ASC, IL-1β, and IL-18, as well as viral load and CRP were observed in HIV-1-infected individuals. Likewise, a significant positive correlation between HDL and CD4+ T-cell counts was found.

**Conclusion:**

In summary, our results indicate that HDL might modulate the expression of several key components of the inflammasomes during HIV-1 infection, suggesting a novel role of HDL in modifying the inflammatory state and consequently, the progression of HIV-1 infection.

## Introduction

Human immunodeficiency virus type 1 (HIV-1), etiological agent of the acquired immunodeficiency syndrome (AIDS), was identified during the early 1980s (1). Despite the implementation of preventive measures, it is currently estimated that 36.7 million people are infected with HIV-1 in the world, so this infection continues to be considered one of the major health problems worldwide (2). The HIV-1 infection is characterized by a gradual increase in viral load, loss of mucosal integrity, increased microbial translocation, and immunologic hyperactivation (3), which is one the main pathogenic mechanism of disease progression (4, 5). It has been reported that several inflammatory markers are increased during HIV-1 infection, including IL-1β, IL-6, IL-8, IL-18, and TNF-α (6–9), as well as others serum markers such as C-reactive protein (CRP), D-dimer, and soluble CD14, among others (10). In fact, *in vitro* studies have reported that IL-1β and IL-18 correlate directly with increased viral replication, which highlights the importance of these pro-inflammatory cytokines in the progression of this infection (11, 12).

In the last years, the importance of the host innate immunity in the initial response to HIV-1 has been recognized (13, 14). Candidate mechanisms responsible for the innate immune responses include stimulation of pattern recognition receptors and the activation of inflammasomes. The inflammasomes are multicomponent complex commonly composed of an NOD-like receptor (NLR), the adaptor protein ASC, and caspase-1, which is required for processing and secretion the active forms of IL-1β and IL-18 (15, 16). The inflammasome activation is a two-step process that requires priming, usually by TLR activation (signal I), which triggers NF-κB pathway, inducing the transcription of pro-IL-1β and pro-IL-18; the second signal, induced by distinct stimuli (16), promotes the assembly of inflammasomes, that in turn endorse the proteolytic activation of these two cytokines (17, 18), contributing to the inflammatory process. Indeed, we have previously shown that during HIV-1 infection, the virus might act as the first signal to activate the NLRP3 inflammasome in macrophages (19) and also that HIV is associated with inflammasome activation (20). In addition, it has been reported that HIV-1 infected dendritic cells, from healthy individuals, exhibit enhanced transcriptional expression of NLRP3, caspase-1, and IL-1β (21), suggesting that HIV-1 has the ability to induce an inflammatory response through these complexes.

Although antiretroviral therapy successfully controls viral load in most patients, it has a minimal effects on the inflammatory status of them (8), underlying the importance of exploring new therapeutic strategies to complement current antiretroviral therapy and improve the quality of life of patients, by decreasing the incidence of comorbidities. In this sense, the immunomodulatory properties of endogenous molecules such as the high-density lipoproteins (HDL) were recently described. Their main protein component is the apoprotein AI (apo-AI), and the lipid fraction is composed of free cholesterol, cholesterol esters, phospholipids, and triglycerides (22, 23). While the main function of HDL is to transport cholesterol from the peripheral tissues to the liver (24), they also have pleiotropic effects in the reduction of inflammation, apoptosis and oxidation of low density lipoprotein (LDL) (25–27). It has been found that HDL neutralize LPS (28), negatively regulate the expression of adhesion molecules such as V-CAM, I-CAM, and E-selectin (29), and modulate the composition of the lipid rafts, important for cell signaling (30) Previously, it was described that HDL can negatively regulate the expression of pro-inflammatory cytokines induced by TLRs triggering, through the transcriptional regulator ATF3 (31). One of the pathways by which HDL mediates their anti-inflammatory effects is the regulation of the inflammasomes activation in response to cholesterol crystals, due to (i) negative regulation of the mRNA expression of NLRP3 and IL-1β, (ii) greater stability of the lysosomal membrane, and (iii) less mitochondrial damage (32). Most studies exploring the immunomodulatory effects of HDL have focused on cardiovascular diseases (33–36); however, the effects on inflammation may be promising in the context of viral infections such as HIV-1, which has an important inflammatory component that defines its progression to AIDS. Other authors have reported metabolic alterations, including lower HDL level in HIV-1-infected patients, when compared with healthy donors (37). Furthermore, the HDL deficiency is a factor that contributes to the high cardiovascular risk observed in patients with HIV-1 (38) and which is associated with the inflammatory process (39). Therefore, the aim of this study was to explore the association between HDL levels and the expression of inflammasome components and other inflammatory markers during HIV-1 infection.

## Materials and Methods

### Population of Study

This is a cross-sectional study, including 36 HIV-1-infected individuals without antiretroviral therapy or active co-infections. Patients with a history of anemia, diabetes, cancer, or thyroid disease, consumption of statins, as well as pregnant women were excluded. As a control group, 36 healthy individuals, matched by age and sex, from the same geographic area were included. The clinical and demographic characteristics of the study participants are presented in Table 1.

**Table 1:** Demographic features of enrolled individuals.

	HIV-1-infected individuals (*n* = 36)	Healthy donors (*n* = 36)
Women:men	17:19	17:19
Age in years: median (IQR)	28 (22.3–37)	30 (24.3–42.5)
CD4+ T-cells/mm^3^: median (IQR)	629 (401–748)	791 (654–1,032)
Viral load copies/mL: median (IQR)	12,552 (1,240–48,368)	N/A
Months since HIV-1 diagnosis: median (IQR)	7 (2–40)	N/A

*IQR, interquartile range, N/A, not applicable; HIV-1, human immunodeficiency virus type 1*.

For statistical analysis, HIV-1-infected individuals were grouped based on: (i) the CD4+ T-cells counts (<200 cells/μL, 200–500 cells/mm^3^, and <500 cells/mm^3^) according to the CDC classification (40); (ii) the viral load (low: <2,000 copies/mL and high: >5,000 copies/mL). The group was also divided according to the CRP levels (normal: <0.5 mg/dL and high: >0.5 mg/dL). Finally, they were classified according to HDL levels (low: <40 mg/dL and normal: >40 mg/dL).

According to ethical guidelines, all subjects signed a written informed consent, previously reviewed and approved by the research ethics committee of the *Universidad Cooperativa de Colombia*. The protocols were carried out in accordance with the principles of the Declaration of Helsinki. All the participants have completed (i) the Alcohol, Smoking and Substance Involvement Screening Test (ASSIST V 3.0) of the World Health Organization to evaluate smoking, alcohol, and other psychoactive substances consumption, (ii) the International Physical Activity Questionnaire, and (iii) a survey of socio-demographic data.

### Sample Taking and Processing

Approximately 20 mL of peripheral blood was obtained by venipuncture in vacutainer tubes. Plasma and serum were isolated by centrifugation, and stored at −70°C until use. Peripheral blood mononuclear cells (PBMCs) were isolated using the Ficoll-Histopaque density gradient method (Sigma-Aldrich Chemical Co., St. Louis, MO, USA).

### Viral Load and CD4+ T-Cells Count in Peripheral Blood

Human immunodeficiency virus type 1 viral load was performed in plasma from peripheral blood samples, by a certified clinical laboratory. CD4+ T-cells counts were determined for all participants to assess their immune status; by flow cytometry (FACS BD Biosciences, San Jose, CA, USA) and using specific monoclonal antibodies (anti-CD3-FITC, clone: UCHT1, anti-CD4-APC; clone: RPA-T4, and anti-CD8-PE, clone: RPA-T8) (eBioscience, San Diego, CA, USA).

### Quantification of HDL and CRP

Serum HDL levels were quantified with a colorimetric assay, using the commercial kit (Biosystems, Costa Brava 30.08030, Barcelona, Spain), the results were reported in milligrams per deciliter. CRP was quantified by a clinical laboratory certified using the immunoturbidimetry.

### Cytokines Quantification by ELISA

Serum levels of IL-1β, IL-6 (BD Biosciences, Franklin Lakes, NJ, USA) and IL-18 (Human IL-18 Matched Antibody Pairs BMS267/2MST, eBioscience, Austria) were evaluated by ELISA using commercial kits, following the manufacturer’s instructions. The samples were processed in duplicate, and the concentration of the cytokines was calculated from a calibration curve.

### Extraction of RNA and cDNA Synthesis

Total RNA was extracted from PBMCs using the commercial RNeasy Mini Kit (QIAGEN Inc., Valencia, CA, USA), following the manufacturer’s recommendations. The RNA concentration/purity was determined by spectrophotometry at 260–280 nm. The cDNA synthesis was performed with 230 ng of total RNA and using the Revertaid™ H Minus First Strand cDNA Synthesis Kit (Fermentas, Glen Burnie, USA), following the manufacturer’s recommendations.

### Quantitative RT-PCR for the Inflammasome Components

The mRNA of NLRP1, NLRP3, NLRC4, AIM2, ASC, caspase-1, IL-1β, and IL-18 was quantified by real-time RT-PCR using Maxima SYBR Green qPCR master mix kit (Fermentas, Glen Burnie, MD, USA). The mRNA β-actin was used as the housekeeping gene to normalize the RNA content. The amplification protocols were 39 cycles and standardized for each gene (41–43). For real-time RT-PCR analysis, the CFX Manager Version: 1.5.534.0511 software (Bio-Rad, Hercules, CA, USA) was used.

### Statistical Analysis

For data analysis, GraphPad Prism^®^ 5.0 (San Diego, CA, USA) software was used. Normality and homoscedasticity were evaluated using Shapiro–Wilk test and Levene test, respectively. Student’s *t*-test or Mann–Whitney test was used for the comparison between groups for parametric and non-parametric data, respectively. Parametric ANOVA or Kruskal–Wallis tests were used for the comparison between three or more groups; in case of statistical association *post hoc* tests (or multiple benchmarks) HDS of Tukey and Dunn, respectively, were carried out. For the correlations, Pearson or Spearman tests were performed, for parametric or non-parametric data, respectively. *p* Values lower than 0.05 were considered statistically significant.

## Results

### Clinical and Demographic Features

The demographic information is presented in Table 1. As expected, HIV-1-infected individuals have a lower CD4+ T-cells count compared with healthy donors (*p* < 0.0001). Regarding physical activity, 23% (HIV-1-infected individuals) and 22% (healthy controls) have low physical activity. Less than 5% of the individuals in both groups have reported moderate levels of alcohol and psychoactive substances consumption.

### HIV-1-Infected Individuals Have Decreased HDL Levels

**Figure 1:**
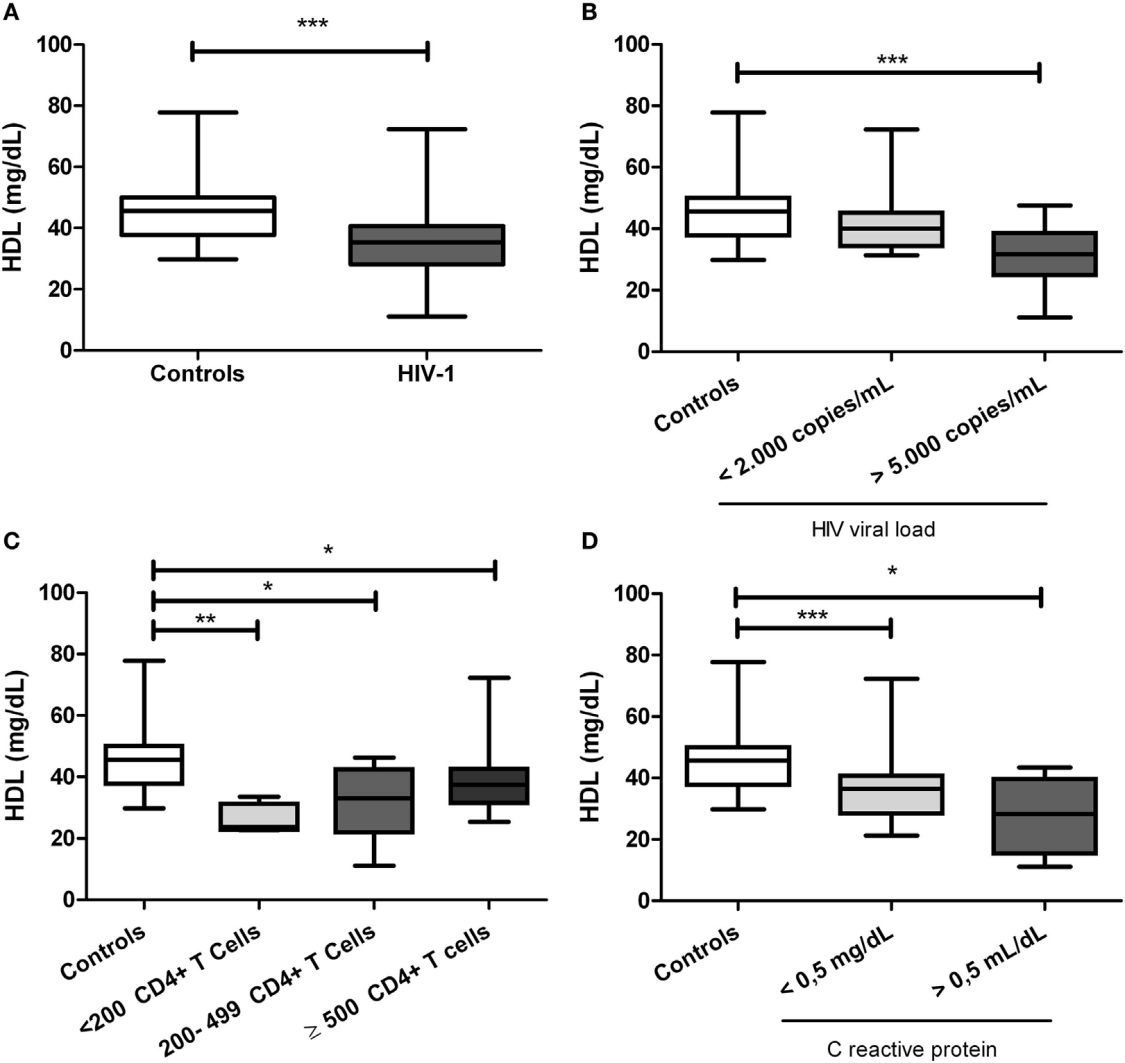
Serum high-density lipoprotein (HDL) levels in human immunodeficiency virus type 1 (HIV-1)-infected individuals and healthy controls. **(A)** HIV-1-infected individuals and healthy controls. In addition, HIV-1-infected individuals were classified according to **(B)** viral load, **(C)** CD4+ T-cells counts, and **(D)** C-reactive protein levels. The statistical comparison was made using Mann–Whitney *U* and Kruskal–Wallis tests with a 95% confidence level; and *post hoc* tests (or multiple benchmarks) HDS of Dunn was made. Significant differences are represented in the upper part of this figure (*p < 0.05; **p < 0, 01; and ***p < 0.0001).

Since several metabolic alterations have been reported during HIV-1 infections (37), the lipid profile was quantified. It was found that HIV-1-infected individuals had significant lower HDL levels compared with healthy controls (*p* < 0.0001, Figure 1A). However, there were not alterations in other lipid profile parameters, including triglycerides, VLDL, LDL, and total cholesterol (data not shown). Moreover, the lower values in HDL were observed in those individuals with viral load over 5,000 copies/mL (*p* < 0.0001, Figure 1B) and also in those patients with CD4+ T-cells counts lower than 200 cells/mm^3^ (*p* < 0.0001; Figure 1C; Figure S1 in Supplementary Material). The decrease in HDL levels is conserved among HIV-1-infected individuals, independently of their CRP levels (Figure 1D). As previously reported (44), women showed higher HDL levels than men, but only within the healthy control group (data no shown). In addition, alterations in HDL levels were no affected by normal or overweight conditions of patients (data no shown).

### The mRNA Expression of Inflammasome-Related Genes Is Altered in HIV-1-Infected Individuals

**Figure 2:**
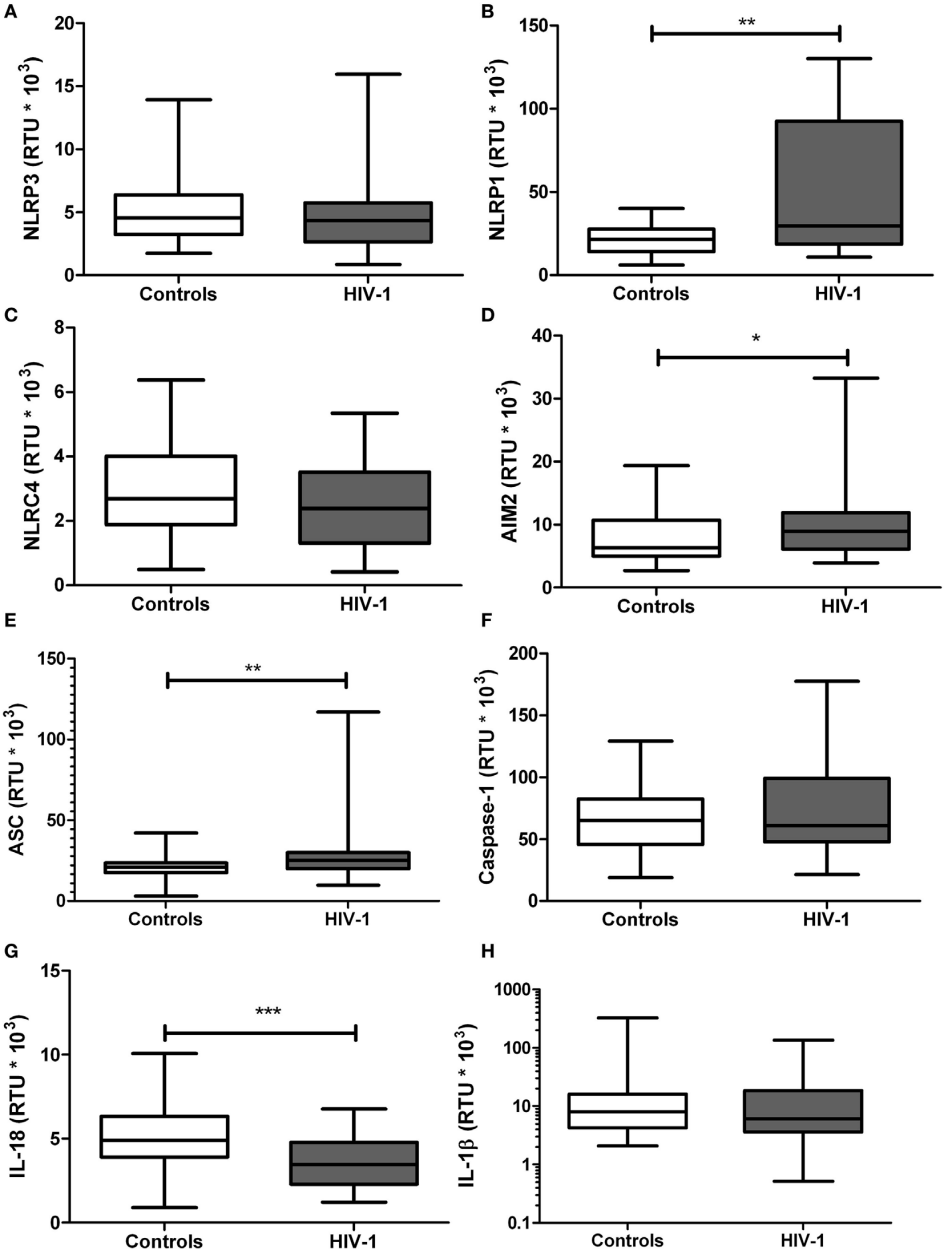
mRNA expression of inflammasome-related genes in human immunodeficiency virus type 1 (HIV-1)-infected individuals and healthy controls. RNA samples obtained from HIV-1-infected individuals and controls were quantified by real-time PCR: **(A)** NLRP3, **(B)** NLRP1, **(C)** NLRC4, **(D)** AIM2, **(E)** ASC, **(F)** caspase-1, **(G)** IL-18, and **(H)** IL-1β. β-Actin was used as the constitutive gene to normalize the RNA content. The statistical comparison was made using the Mann–Whitney *U* test with a 95% confidence level. Significant differences are represented in the upper part of this figure (*p < 0.05; **p < 0, 01; and ***p < 0.0001).

Since the exacerbated inflammatory response is one of the main pathogenic mechanisms of HIV-1 infection, mRNA expression of the inflammasome-related genes was quantified. A significant increase in gene expression of NLRP1 (*p* = 0.0073; Figure 2B), AIM2 (*p* = 0.0455; Figure 2D), and ASC (*p* = 0.0147; Figure 2E) was observed in HIV-1-infected individuals, compared with healthy controls. In addition, a significant decrease in IL-18 gene transcription was seen in HIV-1-infected patients compared with healthy controls (*p* < 0.001; Figure 2G). No significant changes in the mRNA expression were observed for the other evaluated genes, including NLRP3, NLRC4, caspase-1 and IL-1β (Figures 2A,C,F,H, respectively).

### Viral Load Is Related With mRNA Expression of NLRP3 and IL-1β in HIV-1-Infected Individuals

**Figure 3:**
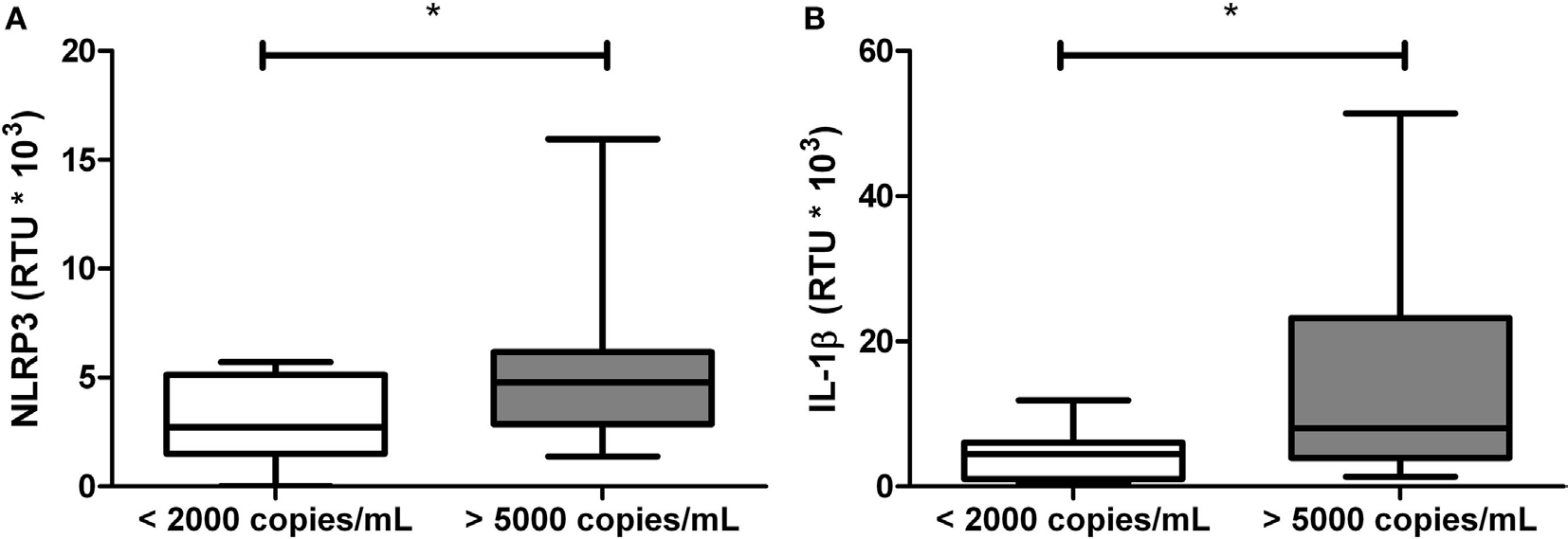
Increased mRNA levels of NLRP3 and IL-1β in patients with high viral loads. Total RNA was obtained from human immunodeficiency virus type 1-infected individuals, classified according to the viral load and quantified by real-time PCR: **(A)** NLRP3 and **(B)** IL-1β. β-Actin was used as the constitutive gene to normalize the RNA content. The statistical comparison was made using the Mann–Whitney *U* with a 95% confidence level. Significant differences are represented in the upper part of this figure (*p < 0.05).

Since viral load, CD4+ T-cells counts, and CRP are related to the inflammatory status and progression in HIV-1-infected individuals, we analyzed whether these variables are related with the gene expression of inflammasome components. Patients with viral loads higher than 5,000 copies/mL were found to have increased expression of NLRP3 and IL-1β compared with those patients with viral loads below 2,000 copies/mL (Figure 3).

### HIV-1-Infected Individuals Show Increases Level of IL-6 and CRP

**Figure 4:**
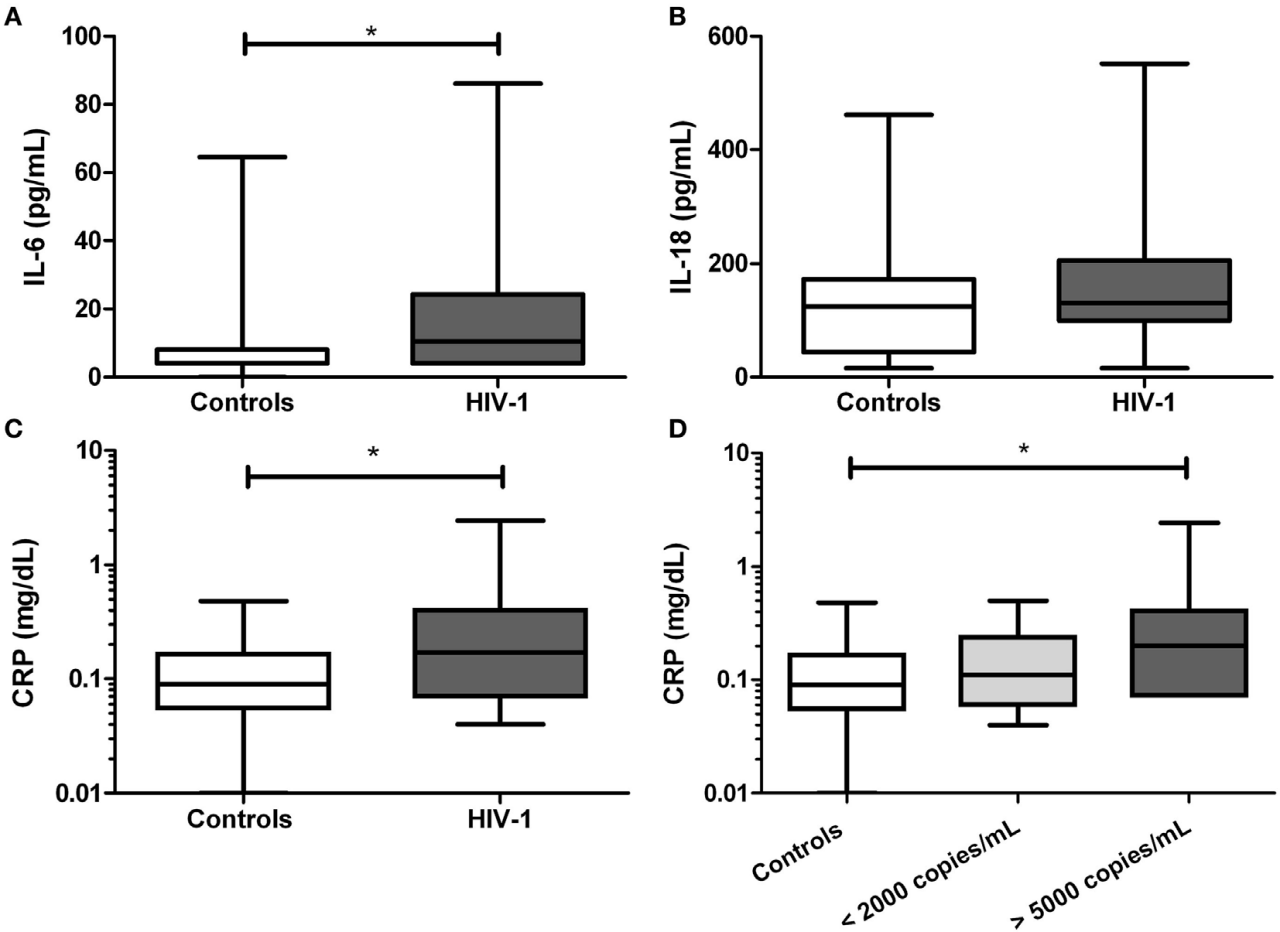
Serum levels of inflammatory markers in human immunodeficiency virus type 1 (HIV-1)-infected individuals. In serum samples from HIV-1-infected individuals and healthy controls were quantified **(A)** IL-6, **(B)** IL-18, and **(C)** C-reactive protein (CRP). In HIV-1-infected individuals, the CRP was compared according to viral load **(D)**. The statistical comparison was made using the Mann–Whitney *U* and Kruskal–Wallis tests with a 95% confidence level and *post hoc* tests (or multiple benchmarks) HDS of Dunn was made. Significant differences are represented in the upper part of this figure (*p < 0.05).

Next, inflammatory markers including IL-1β, IL-18, IL-6, and CRP were evaluated in serum. We found that the IL-6 level was significantly increased in HIV-1-infected individuals compared with healthy controls (*p* = 0.0175, Figure 4A), but no for IL-18 (Figure 4B). The IL-1β was undetectable in all tested subjects. In addition, a significant increase in CRP level (*p* = 0.0125) was observed in HIV-1-infected individuals compared with healthy controls (Figure 4C), and the increase was more evident in those patients with viral load over 5,000 copies/mL (Figure 4D).

### HDL Levels Correlated With mRNA Expression of Inflammasome-Related Genes in HIV-1-Infected Individuals

**Figure 5:**
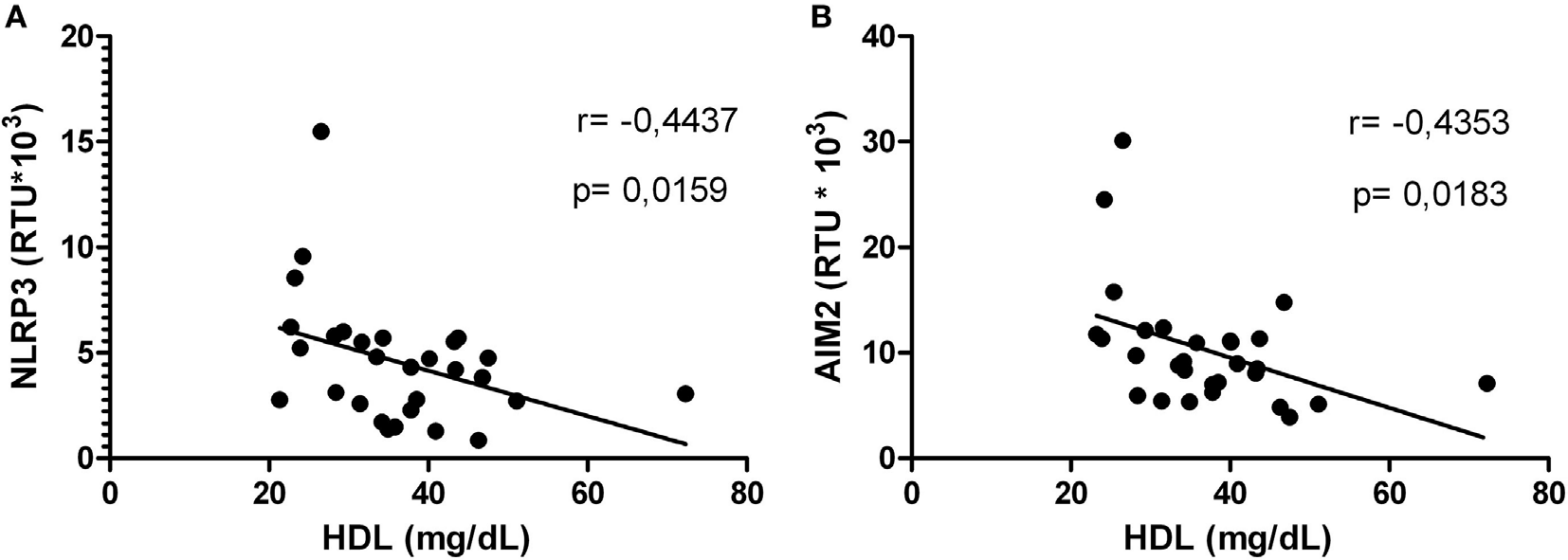
High-density lipoprotein (HDL) level correlated with NLRP3 or AIM2 in human immunodeficiency virus type 1-infected individuals. Correlation between HDL and NLRP3 **(A)** or AIM2 **(B)** was performed using a Spearman test. The *r* and *p* values are indicated in each figure. A *p* value lower than 0.05 was considered as significant.

**Figure 6:**
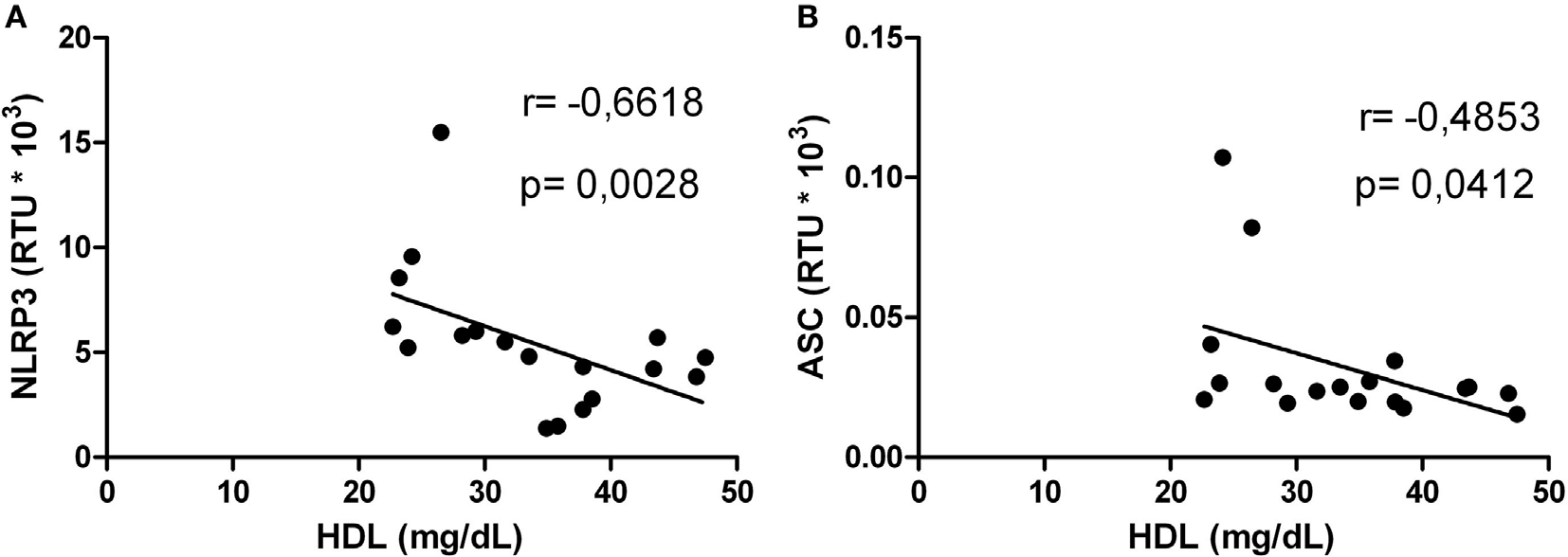
Correlation analysis between high-density lipoprotein (HDL) and NLRP3 or ASC in human immunodeficiency virus type 1-infected individuals. Correlation between HDL and NLRP3 **(A)** or ASC **(B)** in patients with viral load greater than 5,000 copies/mL was performed using a Spearman test. The *r* and *p* values are indicated in each figure. A *p* value lower than 0.05 was considered as significant correlation.

Correlations between HDL levels and the mRNA expression of inflammasome-related genes were explored. Negative correlations were found between HDL and NLRP3 (Figure 5A) or AIM2 (Figure 5B). In addition, HIV-1-infected individuals with viral loads above 5,000 copies/mL showed a negative correlation between HDL and NLRP3 or ASC (Figures 6A,B). Furthermore, it was found in HIV-1-infected individuals with CD4+ T-cells counts greater than 500 cells/mm^3^ a negative correlation between HDL and AIM2 or IL-18 (data no shown).

### HIV-1-Infected Individuals With Normal HDL Levels Exhibit Minor Clinical Progression to Diseases

**Figure 7:**
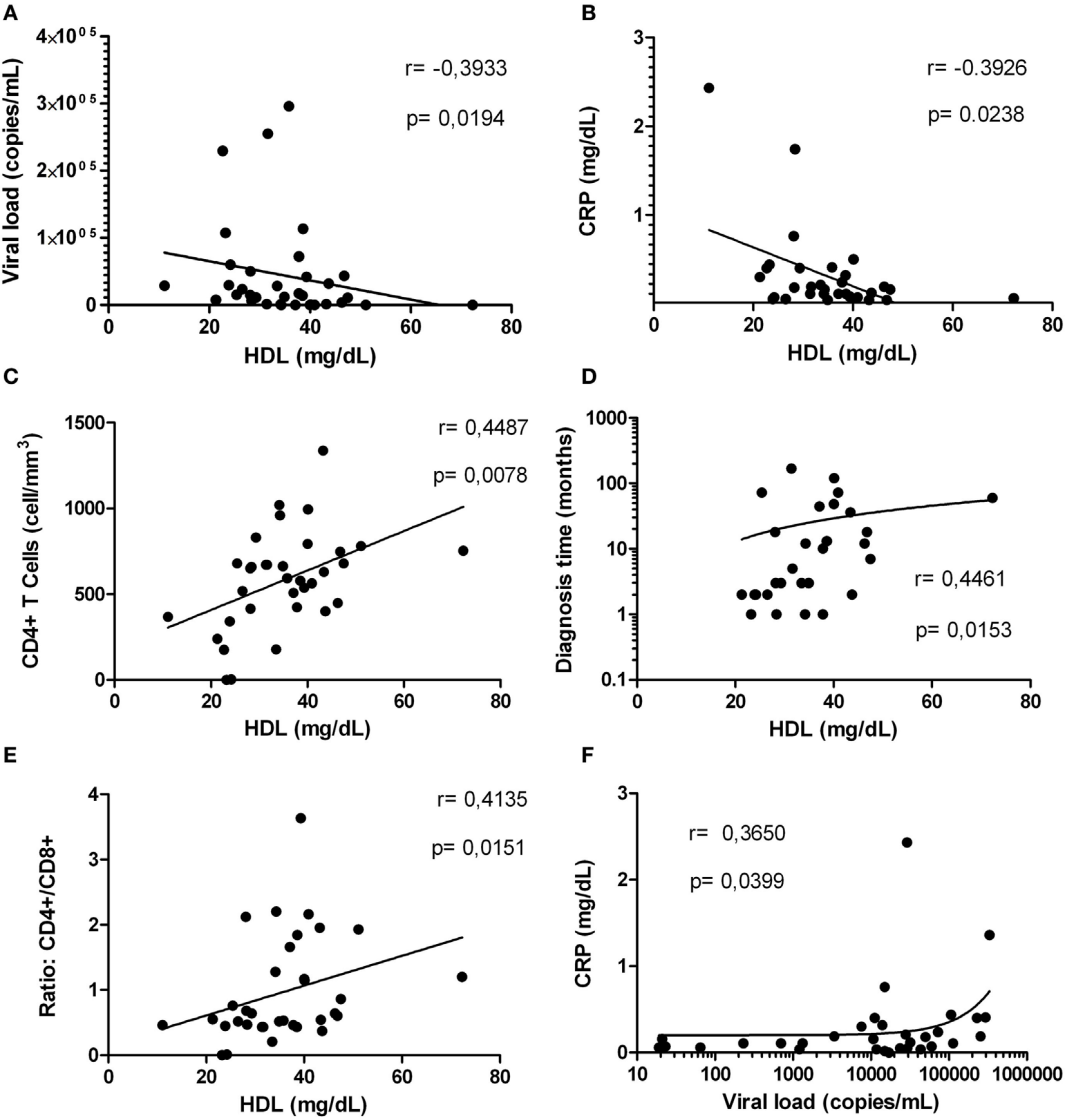
Correlations between high-density lipoprotein (HDL) levels with factors associated with human immunodeficiency virus type 1 progression. Correlation analysis between HDL levels and: viral load **(A)**, C-reactive protein (CRP) **(B)**, CD4+ T-cells **(C)**, diagnosis time **(D)**, and ratio CD4+/CD8+ **(E)**. In addition, correlation between viral load and CRP is presented **(F)**. The correlation analyses were performed using a Spearman test. The *r* and *p* values are indicated in each figure. A *p* value lower than 0.05 was considered as significant correlation.

Based on our results indicating alterations in the inflammatory status and the HDL levels in HIV-1-infected individuals, we proceeded to determine whether HDL correlate with parameters associated with HIV-1 progression. A negative correlation between HDL level and viral load was found (Figure 7A); this observation was most evident in HIV-1-infected individuals with viral loads below 2,000 copies/mL (data no shown). Finally, a negative correlation between HDL levels and CRP levels was demonstrated (Figure 7B).

Finally, since other markers such as CD4+ T-cells count and diagnosis time are important predictors of the HIV-1 disease progression, we assessed whether there was a correlation between HDL levels and these two parameters. Interestingly, HDL levels were positively correlated with CD4+ T-cells count (Figure 7C) and diagnosis time (Figure 7D). These correlations were more evident in HIV-1-infected individuals with viral loads greater than 5,000 copies/mL (data not shown). In addition, a positive correlation between HDL and CD4+/CD8+ T-cells ratio, a predictor of disease progression (45) was found (Figure 7E). Interestingly, the viral load and CRP levels were positively correlated (Figure 7F), as an indicator of the relationship between viral replication and inflammation.

## Discussion

Immune hyperactivation is the major pathogenic mechanism of HIV-1 infection and directly correlated with disease progression, since it leads to immune depletion, loss of viral control, and ultimately to development of AIDS (4, 5). Different inflammatory markers, such as CRP, D-dimer, and pro-inflammatory cytokines, are increased during the course of the infection (6–10). Although the implementation of antiretroviral therapy has led to an increase in the life expectancy of individuals with HIV-1, they continue to present chronic inflammation that is related to various pathological outcomes, such as cardiovascular diseases (46), cancer (47), and renal diseases (48). These conditions underline the importance of searching for immunomodulators that complement antiretroviral therapy, reducing inflammation and thus the comorbidities associated with infection.

Since HDL have immunomodulatory properties (49–52), their study and subsequent use as complementary to antiretroviral therapy would be considered (i.e., pharmacological strategies to increase HDL levels). In the current study, and in agreement with previous reports (37, 53), HIV-1-infected individuals exhibited lower HDL levels than healthy controls (Figure 1A), suggesting that HIV-1 infection leads to an alteration in the metabolism of this lipoprotein. Although no differences were observed in other parameters of the lipid profile, other authors have reported higher levels of triglycerides and a decrease in both, total and LDL cholesterol (49, 50, 54). The apparent discrepancy in the results could be explained by differences among the studied group, in particular related to stages of progression; in addition, the differences show the importance of studies on specific populations, given the heterogeneity in the metabolic and inflammatory profiles.

Pro-inflammatory cytokines have been reported to induce hepatic cholesterol synthesis (55). Based on our results and also in previous data, some questions are raised: (i) Are those lower HDL levels due to sustained inflammatory response in HIV-1-infected individuals? or (ii) Are the lower HDL levels prior HIV-1 infection, a factor that promotes the persistent inflammatory response? These questions underline the importance of further studies to elucidate the causal mechanisms. In accordance to this, it has been reported in HIV-1-infected individuals a negative correlation between HDL levels and inflammatory biomarkers such as neopterin and sTNFR-75, suggesting a relationship between this lipoprotein and immune activation during the infection (56). In fact, according with our previous results (manuscript in preparation) as well as those reported by other authors (25, 26, 57), have shown that HDL have regulatory effects on several components of innate immunity, including the inflammasomes (32), which also have an important role in the pathogenesis of HIV-1 infection. In fact, it has been reported that HIV-1 acts as the primary signal for the activation of the NLRP3 inflammasome in macrophages (19). In addition, polymorphisms in inflammasome-related genes have been associated, not only with increased susceptibility to HIV-1 infection (58, 59) but also with progression to AIDS (59), demonstrating the participation of inflammasomes in the course of HIV-1 infection. Our results indicate alterations in mRNA expression of inflammasome-related genes during HIV-1 infection. In particular, increased mRNA expression of NLRP1, AIM2 and ASC, and decreased expression of IL-18, was observed (Figure 2). Other authors have reported similar results on dendritic cells *in vitro* infected with HIV-1 (21) and *ex vivo* evaluations in microglia and podocytes from HIV-1-infected individuals (60, 61). It is noteworthy that HIV-1 Tat and Vpr proteins have the ability to induce the expression of caspase-1 and IL-1β (60, 62). It should be noted that the patients with higher viral replication display stronger alterations in the inflammasome components; together, these results suggest that HIIV-1 infection promotes the activation of these complexes (19).

The inflammasome activation ends with the active forms of IL-1β and IL-18 that are increased during HIV-1 infection, promoting disease progression (11, 12). Even though the HIV-1 has the ability to induce the expression of IL-1β through TLR8 signaling and inflammasome activation (63), here we were unable to detect IL-1β in sera from HIV-1-infected individuals. This can be explained by the sensitivity of the technique used, since other study reported that serum concentration of IL-1β is very low (8), suggesting the need to use ultrasensitive techniques. In addition, the presence of high levels of IL-1β receptor antagonists such as IL-1RII, previously reported during HIV-1 infection (64), could be responsible for this result, a hypothesis that requires further studies.

Other inflammatory markers reported during HIV-1 infection are IL-6 and the acute-phase reactant CRP; both have been previously correlated with mortality in HIV-1 individuals (65). Taking into account that acute inflammatory responses induce alterations in HDL composition and function (66), and that we found a positive correlation between HIV RNA load and CRP, we postulated that high CRP levels could be related with the decrease of HDL, favoring an inflammatory state. In addition, our results indicate that the increase in CRP occurs mainly in HIV-1-infected individuals with high viral load, suggesting a higher viral replication in the presence of inflammatory response and lower HDL levels. Supporting our results, Thacker et al. reported that HDL mediate anti-inflammatory effects through the regulation of inflammasomes activity (32), among the findings reported, there is a decrease in the gene expression of NLRP3 and IL-1β, as well as high expression of the transcriptional regulator ATF3. This HDL-induced factor has been described as a negative regulator of TLRs signaling and cytokines production (31). In accordance with this, negative correlations between HDL and NLRP3 or AIM2 were observed. The decrease in HDL levels could lead to a reduction in the total cholesterol efflux, resulting in accumulation of cholesterol and the development of cholesterol crystals, which activate the NLRP3 inflammasome (67). In this context, the response mediated by the cholesterol crystals could contribute to chronic inflammation during HIV-1 infection, and hence to disease progression. In fact, our previous results show that HDL decreases the IL-1β production *in vitro*, in response to cholesterol crystals and in fact it was seen a decrease in the size of the crystals of cholesterol in a dose-dependent manner, which could be one of the anti-inflammatory mechanisms exerted by this lipoprotein (manuscript in preparation). These results support our hypothesis that control of HIV-1 progression could be, at least in part, associated with the regulation of inflammasomes by the HDL.

The negative correlations between HDL and the inflammasome components, especially those observed in patients with active viral replication, may indicate that these interactions occur in advanced stages of infection. This could be associated with the immune collapse and progression to AIDS, evidenced by the relationship found between HDL and CD4+ T-cells count or HIV RNA load. Similarly, according to reported by Shen et al. (54), patients with lower CD4+ T-cells counts have lower HDL levels, reinforcing the hypothesis that patients with lower HDL levels could have a higher risk of disease progression.

In addition, it is important to keep in mind that the activity of caspase-1-dependent inflammasomes results in pyroptosis (a type of cell death with release of IL-1β) (68–70), contributing with the elimination of CD4+ T-cells during HIV-1 infection (71). In this context, lower HDL levels could result in increased inflammasomes activation, leading to an increased CD4+ T-cells death, according to the positive correlation between HDL and CD4+ T-cells count observed in this study. In fact, HDL levels have been proposed as a predictor parameter in patients receiving non-nucleoside reverse transcriptase inhibitors (52, 72). On other hand, HDL has antioxidant activity on LDL, which once oxidized (oxLDL) induce the expression of IL-1β, via inflammasomes (67, 73). It has been reported that HDL inhibit the inflammatory response induced by the complement system in response to cholesterol crystals (74, 75), contributing with the regulation of the inflammatory process in HIV-1-infected individuals.

Taken together, our results indicate that during HIV-1 infection there is a decrease in HDL levels along with a persistent inflammatory response, in particular in those individuals with evidence of disease progression (increased HIV RNA load and lower CD4+ T-cells counts). Our results suggest that there is a relationship between HDL and inflammasomes activation during HIV-1 infection, pointing toward new therapeutic strategies based on the ability of this lipoprotein for regulate the activation of these complexes that haven been involved in multi-systemic diseases associated with HIV-1 infection. However, given the complexity of the immune response during HIV-1 infection, more studies are needed to elucidate the precise mechanisms behind these processes, since chronic inflammation may in turn lead to an alteration in HDL composition and hence function, as previously reported in patients with chronic renal dysfunction (76). Moreover, it is needed to conduct new studies in patients under antiretroviral therapy (HAART), since it has been reported that HAART has an impact in lipid levels (77) and in some inflammatory markers (78); to know how this interaction can affect the relation between HDL and inflammasomes and how it could be taking in account in the search of new therapeutic targets.

## Ethics Statement

According to ethical guidelines, all subjects signed a written informed consent, previously reviewed and approved by the research ethics committee of the Universidad Cooperativa de Colombia. The protocols were carried out in accordance with the principles of the Declaration of Helsinki.

## Author Contributions

Funding acquisition and project administration: JH. Conceived and designed the experiments; writing original draft: DM-P, SU-I, and JH. Investigation: DM-P, GC, and JH. Formal analysis: DM-P, JC-A, and JH. Writing review and editing: JC-A, SU-I, and JH. Approval of manuscript for publication: DM-P, GC, JC-A, SU-I, and JH.

## Conflict of Interest Statement

The authors declare that the research was conducted in the absence of any commercial or financial relationships that could be construed as a potential conflict of interest.

## Supplementary Material

Figure S1 Viral load and CD4+ T-cells counts according to high-density lipoprotein (HDL) levels in human immunodeficiency virus type 1 (HIV-1) (HIV-1)-infected individuals. HIV-1-infected individuals were divided according to HDL levels (normal: >40 mg/dL and low: <40 mg/dL) and the viral load **(A)** and the CD4+ T-cells counts **(B)** were analyzed. The statistical comparison was made using the Mann–Whitney *U* tests with a 95% confidence level. Significant differences are represented in the upper part of this figure (**p* < 0.05 and ***p* < 0.01).
