# Supplementary figures and images for: Lower High-Density Lipoproteins Levels During Human Immunodeficiency Virus Type 1 Infection Are Associated With Increased Inflammatory Markers and Disease Progression
Source: Front Immunol. 2018 Jun 14;9:1350. doi: 10.3389/fimmu.2018.01350 (PMC6010517; doi:10.3389/fimmu.2018.01350)

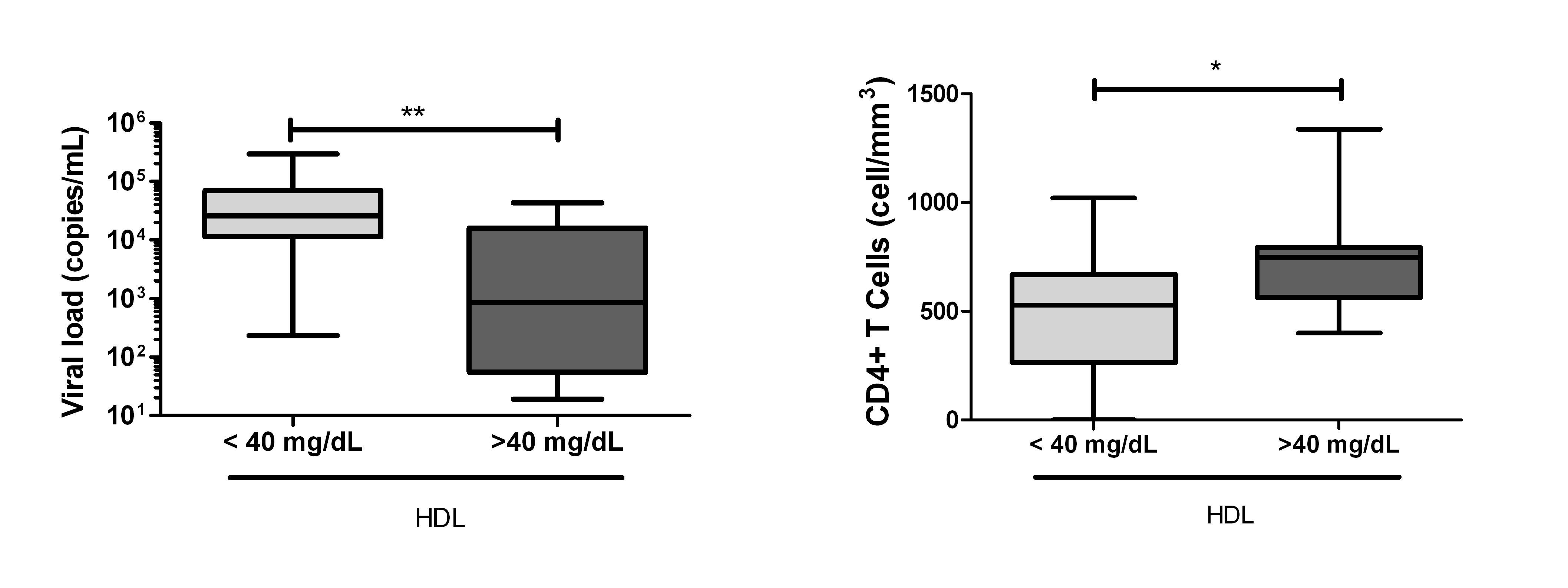

Supplement: Figure S1 — Viral load and CD4+ T-cells counts according to high-density lipoprotein (HDL) levels in human immunodeficiency virus type 1 (HIV-1) (HIV-1)-infected individuals. HIV-1-infected individuals were divided according to HDL levels (normal: >40 mg/dL and low: <40 mg/dL) and the viral load (A) and the CD4+ T-cells counts (B) were analyzed. The statistical comparison was made using the Mann–Whitney U tests with a 95% confidence level. Significant differences are represented in the upper part of this figure (*p < 0.05 and **p < 0.01). [file Image_1.jpeg]
